# Supplementary material for: Sulfated CXCR3 Peptide Trap Use as a Promising Therapeutic Approach for Age-Related Macular Degeneration
Source: Biomedicines. 2024 Jan 22;12(1):241. doi: 10.3390/biomedicines12010241 (PMC10813770; doi:10.3390/biomedicines12010241)
Supplement: Supplementary file 1 [file biomedicines-12-00241-s001.zip › biomedicines-2787303-supplementary.pdf]

Article

Sulfated CXCR3 Peptide Trap Use as a Promising Therapeutic Approach for Age-Related Macular Degeneration

GukHeui Jo [1,†], Jae-Byoung Chae [2,†], Sun-Ah Jung [1], Jungmook Lyu [3], Hyewon Chung [2,*] and Joon H. Lee [1,*]

1 Myung-Gok Eye Research Institute, Kim's Eye Hospital, Konyang University College of Medicine, Seoul 07301, Republic of Korea
2 Department of Ophthalmology, Konkuk University College of Medicine, Seoul 05029, Republic of Korea
3 Department of Medical Science, Konyang University, Seo-gu, Daejeon 35365, Republic of Korea
* Correspondence: joonhlee@konyang.ac.kr (H.C.); hchung@kuh.ac.kr (J.H.L.); Tel.: +82-2-2637-7871 (H.C.); 82-2-2030-7657 (J.H.L.)
† These authors contributed equally to this study.

Supplementary Material

Supplementary Figures

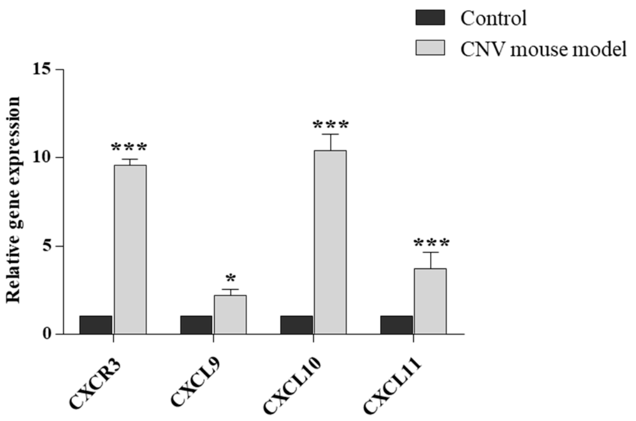

Figure S1. Quantification of mRNA expression of CXCR3 and its ligands (CXCL9, CXCL10, and CXCL11) in the RPE/choroid complex from a control group and a laser-induced CNV mouse model (n=3). *** $p < 0.001$, * $p < 0.05$.

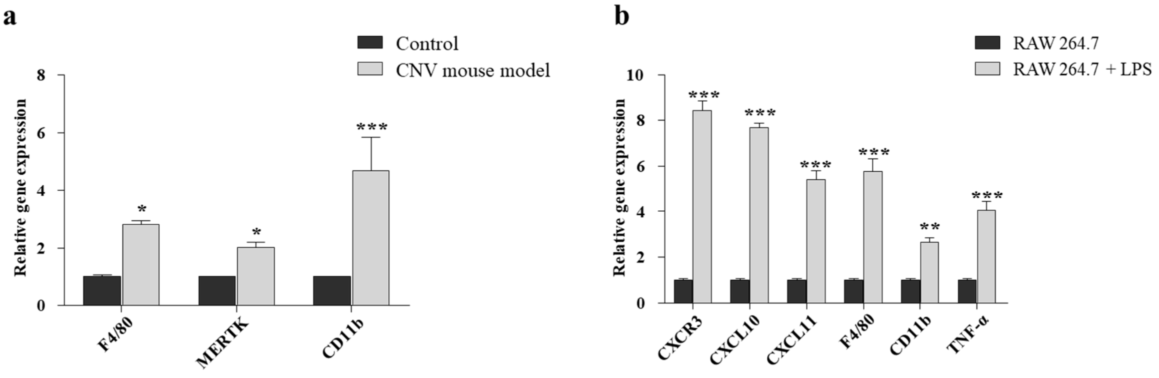

Figure S2. Quantification of mRNA expression of markers related to macrophages in mouse RPE/choroid and RAW 264.7 cells. (a) mRNA expression of macrophage related genes, including F4/80, MERTK and CD11b were increased in RPE/choroid complex from a laser-induced CNV model compared to a control group (n=3). (b) Quantification of mRNA expression in RAW 264.7 cells. In LPS-treated group, the expression of CXCR3, its ligands, and macrophage related genes has increased compared to control group (n=3). *** $p < 0.001$, ** $p < 0.01$, * $p < 0.05$.

Supplementary Table

Table S1. CXCR3 primers used in oligo shuffling. In total, 10 oligos (39 nt in length) were synthesized.

Primer	Sequence (5′ to 3′)
hCXCR3	1F: aaa gat atc atg gtc ctt gag gtg agt gac cac caa gtg
	2F: ctt aat gac gcc gag gtt gcc gcc ctc ctg gag aac ttc
	3F: agc tct tcc tat gac tat ggt gaa aac gag agt gac tcg
	4F: tgc tgt acc tcc ccg ccg tgc cca cag gac ttc agc ctg
	5F: aac ttc gac cgc ctc gag aaa aaa aaa aaa aaa aaa aaa
	3F sulfation: agc tct tcc tag gac tag ggt gaa aac gag agt gac tcg
	1R: ttt ctc gag gcg gtc gaa gtt cag gct gaa gtc ctg tgg
	2R: gca cgg cgg gga ggt aca gca cga gtc act ctc gtt ttc
	3R: acc ata gtc ata gga aga gct gaa gtt ctc cag gag ggc
	4R: ggc aac ctc ggc gtc att aag cac ttg gtg gtc act cac
	5R: ctc aag gac cat gat atc ttt ttt ttt ttt ttt ttt ttt
	3R sulfation: acc cta gtc cta gga aga gct gaa gtt ctc cag gag ggc
mCXCR3	1F:aaa gga tcc atg tac ctt gag gtt agt gaa cgt caa
	2F:gtg ctt gat gcc tcg gac ttt gcc ttt ctt ctg gaa aac
	3F: agc acc tct ccg tac gat tat ggc gaa aac gag agc gac
	4F:ttc tct gac tcc ccg ccg tgc cca cag gat ttc agc
	5F:ctg aac ttt gac cgt ctc gag ttt ttt ttt ttt ttt ttt
	3F sulfation: agc acc tct ccg tag gat tag ggc gaa aac gag agc gac
	1R: aaa ctc gag acg gtc aaa gtt cag gct gaa atc ctg tgg
	2R: gca cgg cgg gga gtc aga gaa gtc gct ctc gtt ttc gcc
	3R: ata atc gta cgg aga ggt gct gtt ttc cag aag aaa ggc
	4R: aaa gtc cga ggc atc aag cac ttg acg ttc act aac
	5R: ctc aag gta cat gga tcc ttt ttt ttt ttt ttt ttt ttt
	3R sulfation: cta atc cta cgg aga ggt gct gtt ttc cag aag aaa ggc

Table S2. Sequence of CXCR3 amino acid and prtein estimated size.

Protein	Type	Amino acid sequence	size
hCXCR3	wt	MYLEVSERQVLDASDFAFLLENSTSPYDYGENESDFSDSPPCPQDF SLNFDR (52)	5.72 kDa
	sulfation	MYLEVSERQVLDASDFAFLLENSTSPYDYGENESDFSDSPPCPQDF SLNFDR (52)	5.72 kDa
mCXCR3	wt	MVLEVSDHQVLNDAEVAALLENFSSSYDYGENESDSCCTSPPCPQ DFSLNFDR (53)	5.83 Kda
	sulfation	MVLEVSDHQVLNDAEVAALLENFSSSYDYGENESDSCCTSPPCPQ DFSLNFDR (53)	5.83 Kda

Red: Sulfation site.

Table S3. List of primers used in PCR reactions.

Gene	Forward primer (5′-3′)	Reverse primer (5′-3′)
**CXCR3**	TACGATCAGCGCCTCAATGCCA	AGCAGGAAACCAGCCACTAGCT
**CXCL9**	CCTAGTGATAAGGAATGCACGATG	CTAGGCAGGTTTGATCTCCGTTC
**CXCL10**	ATCATCCCTGCGAGCCTATCCT	GACCTTTTTTGGCTAAACGCTTTC
**CXCL11**	CCGAGTAACGGCTGCGACAAAG	CCTGCATTATGAGGCGAGCTTG
**F4/80**	GCTCTCCATCTGCCTTTTCCTG	GGAAGCCAAGTAGAGGTAGTGC
**MERTK**	ATCATCCTCGGCTGCTTCTGTG	ACGACCAGTTGGGAATCCTCCT
**CD11b**	TACTTCGGGCAGTCTCTGAGTG	ATGGTTGCCTCCAGTCTCAGCA
**TNF-α**	GGTGCCTATGTCTCAGCCTCTT	GCCATAGAACTGATGAGAGGGAG
